# Social learning and exploration–exploitation dilemma in decision-making

**DOI:** 10.3389/fncir.2026.1781811

**Published:** 2026-03-02

**Authors:** Gota Morishita, Shinsuke Suzuki

**Affiliations:** 1 Centre for Brain, Mind and Markets, The University of Melbourne, Parkville, VIC, Australia; 2 Faculty of Social Data Science, Hitotsubashi University, Kunitachi, Japan; 3 Brain Research Center, Hitotsubashi Institute for Advanced Study, Hitotsubashi University, Kunitachi, Japan

**Keywords:** computational model, decision-making, imitation, observational learning, reinforcement learning, reward, social cognition

## Abstract

This mini review examines the neurocomputational principles of social learning through the lens of the exploration–exploitation dilemma. While the neural mechanisms of learning from others—mediated by distinct signals in the ventromedial and lateral prefrontal cortices—are well established, less is known about how these mechanisms interact with the fundamental trade-off between gathering information (“exploration”) and maximizing rewards (“exploitation”). We discuss how social environments shape this trade-off, leading to strategic behaviors such as informational free-riding or conformity. A central focus of this review is the issue of source selection: how agents decide whom to observe. We present recent evidence suggesting that, contrary to the predictions of optimal information-seeking theories, humans often exhibit a “reliability-seeking” bias, preferring to learn from consistent, exploitation-oriented partners rather than highly exploratory ones. We conclude by discussing the limitations of current paradigms, specifically the inherent confounding of social cues such as competence and predictability, and outline a computational framework for isolating the specific drivers of adaptive social decision-making.

## Introduction

**Figure 1:**
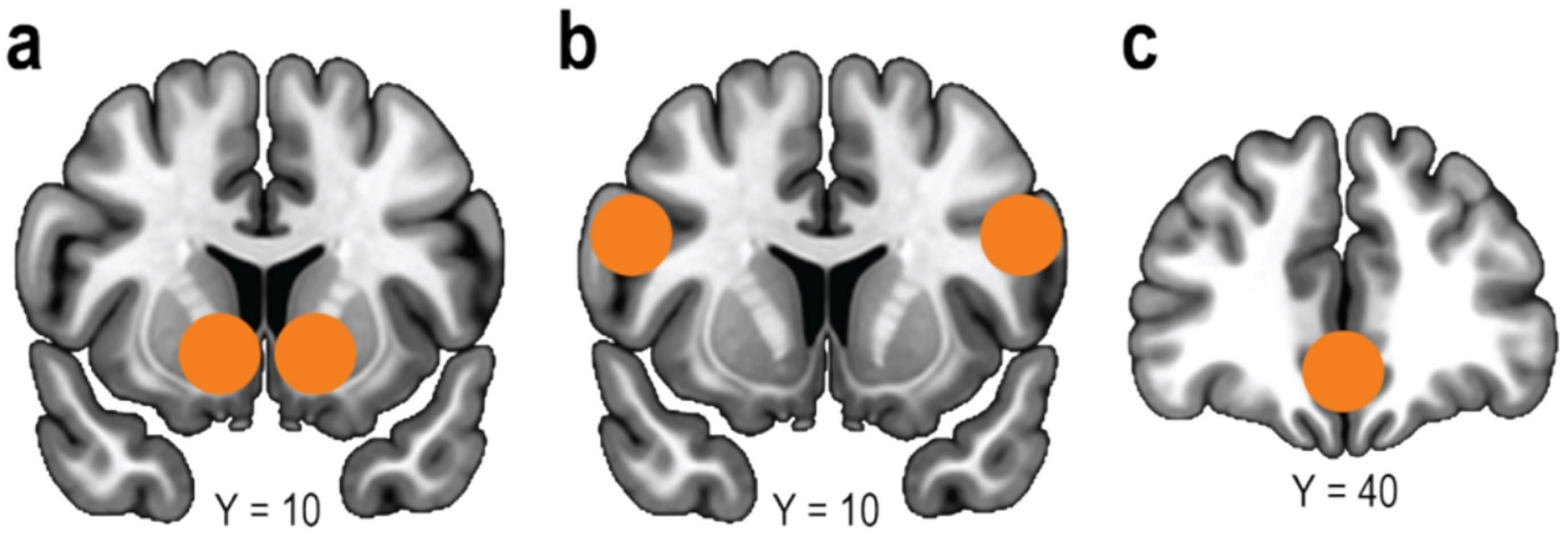
Brain regions implemented in individual and social learning. **(a)** Ventral striatum. **(b)** Lateral prefrontal cortex (lPFC). **(c)** Ventromedial prefrontal cortex (vmPFC).

Adaptive decision-making to attain rewards is fundamental for animal survival. The reinforcement learning framework posits that individuals can acquire optimal behaviors that maximize cumulative rewards through trial and error (Sutton and Barto, 1998). This learning is formalized as updating the value of a choice option based on the reward prediction error, defined as the discrepancy between the obtained reward and the current value of the chosen option. Furthermore, the reward prediction error is known to be encoded by dopaminergic neural activity (Schultz et al., 1997; Glimcher, 2011). In humans, the error signal is correlated with the blood-oxygenation-level-dependent (BOLD) signals in the ventral striatum (Figure 1a), a major projection site of dopamine (O’Doherty et al., 2004; Rutledge et al., 2010). This framework is highly influential, as it successfully captures both behavior and neural activity of animals, including humans (Rangel et al., 2008).

Individuals can learn not only from direct experience but also from the experiences of others—a process known as social learning (Olsson et al., 2020; Gariépy et al., 2014; Behrens et al., 2009). For example, Adélie penguins utilize social learning to assess predation risk before foraging (Chamley, 2004). Rather than diving immediately, the group waits to observe the outcome of the first penguin’s entry. If no predator appears, the others follow, inferring that the environment is safe. Analogously, humans rely on social learning in value-based decision-making. When selecting a restaurant, for instance, individuals often consult online reviews or peer recommendations. This leverages others’ experiences to identify high-quality ones while mitigating the risk of a poor outcome. Thus, social learning supports adaptive decision-making by enabling individuals to acquire optimal behaviors without relying solely on direct trial and error.

In this mini review, we discuss recent findings on the computational principles of social reinforcement learning. In particular, we focus on two under-explored issues: from whom individuals choose to learn, and how they adjust their social learning strategies to navigate the “exploration–exploitation dilemma,” a key computational challenge in reinforcement learning. We believe that this review offers a new perspective on the social learning literature by re-examining it through the lens of this fundamental reinforcement learning challenge.

### Exploration–exploitation dilemma in individual learning

Before addressing social learning, we first characterize the exploration–exploitation dilemma in individual contexts (Schulz and Gershman, 2019; Wilson et al., 2021; Hills et al., 2015). This dilemma is best illustrated by the “restaurant problem.” Consider moving to a new city and searching for good dining options. After a period of trial and error, you identify a favorite. At that point, you face a choice: return to your favorite place (“exploitation”) or continue searching for potentially better options (“exploration”). If you explore too much, you forego the guaranteed pleasure of your favorite spot; if you exploit too much, you risk missing out on a superior experience. Due to this inherent trade-off, the problem is non-trivial. While computer science has proposed various algorithms to address it (Lattimore and Szepesvári, 2020), no single definitive solution exists for all environments.

**Figure 2:**
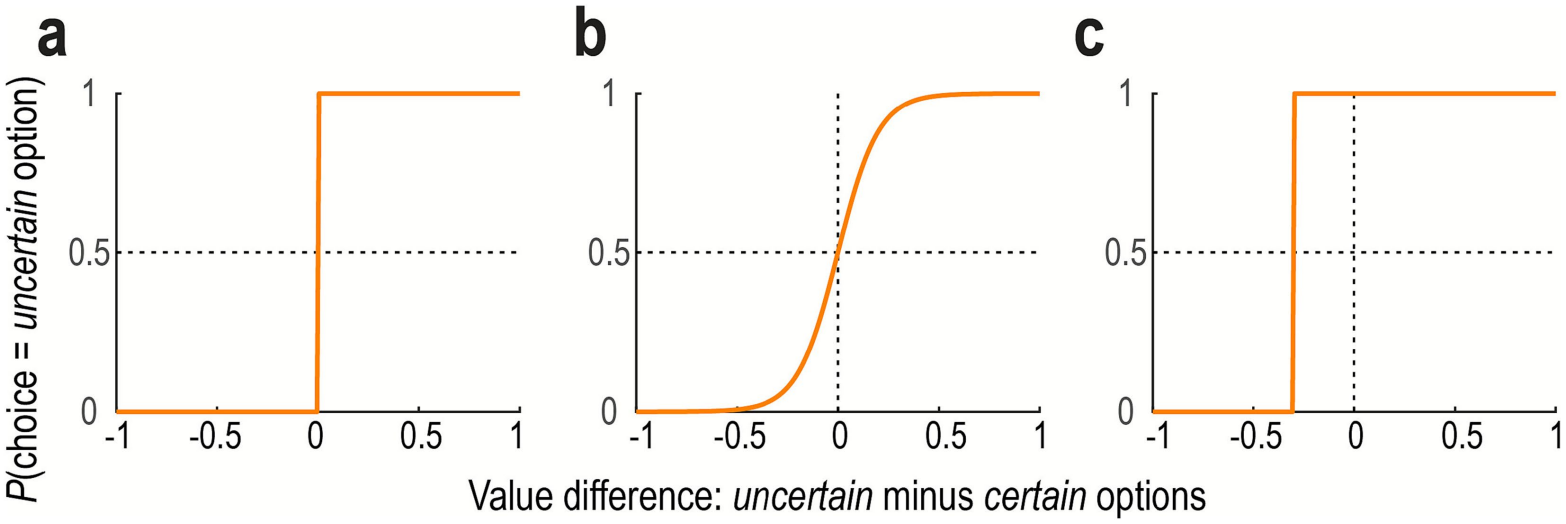
Decision-making with different exploration strategies. **(a)** No exploration. The probability of choosing the uncertain option over the certain option is plotted as a function of the value difference. The agent always chooses the option with the higher estimated value (i.e., deterministic). **(b)** Random exploration. The agent generally favors the option with the higher value, but with added stochasticity (noise). Consequently, the lower-value option is occasionally chosen by chance. The format is the same as **(a)**. **(c)** Directed exploration. The agent considers the uncertainty of the value estimation. The agent is more likely to choose the uncertain option, even if its expected value is lower, to gain information. The format is the same as **(a)**.

A simple algorithm is called “random exploration.” In this algorithm, exploration is implemented as stochasticity in decision-making. That is, an agent sometimes chooses the option with a lower estimated value with a certain probability, which is exploratory behavior. A more sophisticated version is called “directed exploration.” In this algorithm, an agent takes into account the uncertainty of value estimation for each of the available options, often defined by the Bayesian precision or approximated by the number of times the agent sampled the corresponding option. That is, the agent is more likely to choose an uncertain (unfamiliar) option. Consider the case of decision-making with two alternative options. If the agent exhibits no exploration, her choice is guided by the value difference without noise (Figure 2a). If she employs random exploration to some degree, she sometimes chooses the option with lower value (Figure 2b). In directed exploration, her choice is guided not only by the value difference but also by the uncertainty of value estimation (Figure 2c): the uncertain option is more likely to be chosen.

In neuroscience and psychology, a growing body of work has investigated how humans resolve the exploration–exploitation dilemma. Behavioral studies indicate that people deploy at least two dissociable strategies (Gershman, 2018; Wilson et al., 2014): random exploration, which increases choice stochasticity to sample alternatives, and directed exploration, which preferentially targets informative options (e.g., those with higher uncertainty). Developmental evidence suggests that these two forms of exploration follow distinct developmental trajectories, implying separable underlying mechanisms (Somerville et al., 2017). Computational modeling of behavior further demonstrates that people adjust the extent of the two types of exploration depending on uncertainty in their value estimates, in a manner consistent with Thompson sampling and the Upper Confidence Bound (UCB) algorithm (Gershman, 2018). Moreover, neuroimaging and brain stimulation studies have linked directed exploration to neural computations in the ventrolateral prefrontal cortex (vlPFC), whereas random exploration has been associated with the dorsolateral prefrontal cortex (dlPFC) (Tomov et al., 2020; Zajkowski et al., 2017; Badre et al., 2012). Together, these findings support a neurocomputational account in which multiple exploration systems—implemented in distinct prefrontal circuits—jointly support adaptive decision-making.

### Neurocomputational mechanisms of human social learning

A large body of research has examined the neurocomputational mechanisms underlying human social learning by combining functional magnetic resonance imaging (fMRI) with computational modeling (Charpentier and O’Doherty, 2018; Burke et al., 2010). These studies have demonstrated that social learning is not a unitary process but relies on multiple complementary strategies implemented in distinct subregions of the prefrontal cortex (PFC) (Suzuki and O’Doherty, 2020).

The first strategy is learning from others’ rewards (often termed “emulation” of value). In this process, observers update their own value estimates of options by monitoring the outcomes received by others. This learning is driven by observational reward prediction errors—the discrepancy between the observed reward and the observer’s expectation. Crucially, neuroimaging evidence consistently localizes this signal to the ventromedial prefrontal cortex (vmPFC) (Figure 1c) (Burke et al., 2010; Suzuki et al., 2012), a region central to personal value processing. A meta-analysis has further confirmed that the vmPFC encodes reward prediction errors regardless of whether the recipient is the self or another, suggesting a shared neural representation for value updating (Morelli et al., 2015).

The second strategy is learning from others’ actions (“imitation”). Unlike emulation, this process focuses on predicting the partner’s behavior itself, independent of the immediate outcome. This form of learning is driven by action prediction errors—the discrepancy between a partner’s actual choice and the observer’s prediction of that choice. Such signals are typically associated with activity in the lateral PFC (lPFC; Figure 1b), a region implicated in inferring others’ intentions or hidden states (Burke et al., 2010; Suzuki et al., 2012). Collectively, these findings suggest that these two distinct strategies, emulation and imitation, shape social learning.

Findings on the striatal roles in social learning to date are mixed. Some studies have reported significant coupling between observational reward prediction errors and neural activity in the dorsal and ventral striatum (Cooper et al., 2012; Burke et al., 2010), consistent with the striatum as a core neural locus of emulation. However, a meta-analysis did not implicate the striatum in encoding observational prediction errors (Morelli et al., 2015).

### Modulation of social learning strategies

In social learning settings, individuals can free-ride on others’ exploration. By observing the outcomes of others’ choices, people can acquire novel information about unfamiliar options without sampling those options themselves. Crucially, learning from others’ exploration allows individuals to avoid the direct costs of exploration (i.e., foregoing the immediate benefits of exploiting the currently best-known option). Theoretical studies in economics have shown that such informational externalities can generate a free-rider problem when extending the individual reinforcement learning framework (i.e., the two-armed bandit task) to a multi-player setting (Bolton and Harris, 1999; Keller et al., 2005; Rogers, 1988; Suganuma et al., 2025). Specifically, when information produced by exploration is non-excludable, rational agents may strategically reduce their own exploration, relying instead on others to bear the cost. Consequently, the aggregate level of exploration falls below the socially optimal level. These mathematical analyses demonstrate that social learning does not necessarily yield socially ideal outcomes and can sometimes lead to a stagnation of exploration.

Empirical evidence regarding these predictions, however, remains mixed. Several studies utilizing a multi-player reinforcement learning task have observed a reduction in exploration (Toyokawa et al., 2014; Witt et al., 2024). In this set of experiments, participants exhibited lower levels of random and directed exploration in group contexts compared to individual contexts—a pattern consistent with strategic free-riding, even while collective performance improved. In contrast, a recent study reported social conformity in exploration (Danwitz and von Helversen, 2025). In their experiment, participants performed a task alongside agents exhibiting varying degrees of directed exploration. The results showed that exposure to highly exploratory others led participants to increase their own random and directed exploration. This suggests that social information can promote, rather than suppress, exploratory behavior. Together, these findings highlight that social learning can either attenuate or amplify exploration depending on the task structure and the observed behavior.

Beyond contexts focused specifically on exploration, a substantial literature has examined how people adapt their social learning strategies based on the characteristics of others (Najar et al., 2020; Kang et al., 2024; Kang et al., 2021). For example, one study tested whether individuals modulate the degree of imitation—learning from others’ actions—depending on the quality of the social source. Computational modeling indicated that imitation is selectively upregulated when learning from high-performing others, consistent with the idea that observers weight social information by its inferred reliability (Najar et al., 2020). Additionally, a study combining behavioral modeling with continuous theta-burst stimulation (cTBS) probed the neural mechanisms governing when imitation is deployed. Results suggest that imitation is prioritized when others’ actions are predictable, and that this predictability-dependent reliance is causally regulated by the dorsomedial prefrontal cortex (dmPFC) (Kang et al., 2024). Social learning is also shaped by group membership; for instance, imitation typically increases for in-group relative to out-group members (Kang et al., 2021). Notably, individual differences in this bias are captured by neural learning signals: the differential weighting of in-group information correlates with action prediction error encoding in the lateral prefrontal cortex (lPFC), suggesting that lPFC computations support selective updating based on social identity (Kang et al., 2021). An open question is whether such social learning strategies rely on social-specific or domain-general mechanisms (Heyes and Pearce, 2015). Although a recent behavioral study suggests that social learning strategies are updated via domain-general associative learning (Schultner et al., 2025), further evidence is needed to draw a definitive conclusion and to clarify the underlying neural mechanisms.

Recent work has explored how the brain arbitrates between imitation and emulation. For instance, the ventral PFC has been shown to dynamically control the weights assigned to imitation versus higher-order emulation of others’ goals on a trial-by-trial basis, prioritizing the strategy that offers greater predictive reliability (Charpentier et al., 2020). A subsequent study further revealed that individual differences in reliance on higher-order emulation were associated with autism-like traits in the general population (Wu et al., 2024).

Another critical factor modulating social learning strategies is the observer’s own decision confidence. From a Bayesian perspective, optimal information integration requires weighing sources according to their reliability. Consistent with this, a breadth of experimental work demonstrates that individuals rely more heavily on social information when their own estimation of the environment is uncertain—a strategy often referred to as “copy-when-uncertain” (Morgan et al., 2012). Specifically, when subjective confidence in one’s own choice is low, the weight assigned to social signals increases, effectively acting as a compensatory mechanism (Pescetelli et al., 2021; Danwitz and von Helversen, 2025; Morgan et al., 2012; Toyokawa et al., 2017). However, this uncertainty-dependent modulation is not uniform across the population; substantial individual differences exist in how strictly agents adhere to this optimal weighting, with some individuals exhibiting persistent egocentric biases regardless of their own uncertainty (Danwitz and von Helversen, 2025; Toyokawa et al., 2017; Morin et al., 2021).

### Partner selection in social learning

A relatively underexplored issue in social learning is partner selection. In typical laboratory experiments, participants are assigned a fixed partner and learn from the partner’s experiences; they rarely have the opportunity to choose whom to observe. In real-world settings, however, individuals actively select their information sources. For example, when choosing a restaurant based on social media, one must decide whose opinions to trust. Such selection decisions can fundamentally shape the efficacy of social learning.

Our recent study examined whom people prefer to learn from, framing the question in terms of the exploration–exploitation dilemma (Morishita et al., 2025). Using a behavioral experiment combined with computational modeling, we tested two competing hypotheses. The first hypothesis posits that individuals preferentially learn from partners exhibiting a higher degree of random exploration. This strategy would be advantageous because the partner’s exploration generates new information, allowing the learner to continue exploiting currently favorable options. The second hypothesis posits that individuals preferentially learn from partners with a lower degree of random exploration. This strategy would be advantageous when learners primarily rely on imitation (i.e., learning from others’ actions), as less exploratory partners behave more consistently and may therefore appear more successful and reliable.

The preregistered experiment supported the reliability-seeking hypothesis: participants exhibited a significant preference for learning from less exploratory partners over highly exploratory ones. Furthermore, subsequent computational analyses revealed that individual differences in this partner preference were linked to specific social learning styles. Participants who preferred less exploratory partners relied primarily on imitation (learning from others’ actions), whereas those who preferred highly exploratory partners relied more on emulation (learning from others’ rewards). These findings suggest that while there is a general bias toward stable, reliable partners, this preference is modulated by the observer’s underlying learning strategy: imitators seek consistency, while emulators seek information.

Research indicates that partner selection in social learning is governed by multiple factors. First, people preferentially learn from successful individuals. For example, in an artifact-design task, participants imitated peers who achieved higher payoffs, consistent with a “success-biased” strategy (Mesoudi, 2011). Related work suggests that learners also copy “prestigious” individuals—defined as those who have been frequently copied by others in the past (Brand et al., 2020)—indicating that social influence is amplified by reputational cues beyond objective performance. Analogously, there is a robust tendency toward social conformity: following the majority. Across diverse paradigms—ranging from perceptual and value-based decision-making (Suzuki et al., 2015, 2016; Garvert et al., 2015; Reiter et al., 2019) to reinforcement learning (Burke et al., 2010; Charpentier et al., 2020)—individuals systematically shift their choices toward group norms. Similar effects appear in preferential domains like food evaluation and face attractiveness (Klucharev et al., 2009; Nook and Zaki, 2015; Levorsen et al., 2021). Together, these findings demonstrate that partner selection is shaped by multiple sources of influence: demonstrated success, socially conferred prestige, and majority norms.

A promising future direction is to investigate the neurocomputational mechanisms underlying reliability-seeking biases in partner selection—preferences to learn from partners who are competitive, predictable, successful, and/or in the majority. Prior studies using a range of decision-making tasks in social contexts have implicated the medial prefrontal cortex (mPFC) and temporoparietal junction (TPJ) in tracking others’ expertise (Wittmann et al., 2016; Boorman et al., 2013), fidelity (Behrens et al., 2008), and the majority’s choice (Suzuki et al., 2015). Furthermore, predictability-dependent social learning has been shown to be causally regulated by the dmPFC (Kang et al., 2024). These results suggest that a network including the mPFC and TPJ may govern reliability-seeking preferences.

## Discussion

In this mini review, we summarize recent advances in the neurocomputational principles of social learning. In particular, we discussed how social learning strategies—including the critical decision of from whom to learn—are modulated by partner characteristics such as the exploration–exploitation balance, decision quality, predictability, group membership, and social status. However, a significant open question remains: which specific partner attributes drive these strategic adjustments? In naturalistic settings, these characteristics are often empirically intertwined. For example, a lower level of random exploration (i.e., reduced noise) typically correlates with higher decision quality, greater predictability, and often prestige or majority status. Similarly, in-group membership frequently covaries with predictability, as agents possess richer prior knowledge about their own group’s norms. Consequently, it is difficult to determine whether observed biases reflect sensitivity to competence, predictability, social identity, or a combination thereof, while a supplementary analysis in our study suggests that competence contributes more than predictability (Morishita et al., 2025). Furthermore, few studies in the social learning literature to date have carefully distinguished between random and directed exploration (but see Danwitz and von Helversen, 2025). Future work should therefore rigorously test the primary contribution of competence and/or disentangle these factors—for example, by orthogonalizing competence, predictability, and status in experimental designs—and develop computational models that separately parameterize beliefs about a partner’s reliability versus their informational value. Such precision is essential to clarify how social learning is adaptively tuned and which neural computations implement these adjustments.
